# Risk factors for hypertensive disorders of pregnancy among mothers in Tigray region, Ethiopia: matched case-control study

**DOI:** 10.1186/s12884-018-2106-5

**Published:** 2018-12-06

**Authors:** Hailemariam Berhe Kahsay, Fikre Enquselassie Gashe, Wubegzier Mekonnen Ayele

**Affiliations:** 1 School of Nursing, Mekelle University, P.O.Box:1871, Mekelle, Ethiopia; 2 School of Public Health, Addis Ababa University, Addis Ababa, Ethiopia

**Keywords:** Hypertensive disorders of pregnancy, Gestational hypertension, Preeclampsia, Tigray, Ethiopia

## Abstract

**Background:**

Hypertensive disorders of pregnancy are a global public health concern both in developed and developing countries. However, evidences regarding the risk factors of hypertensive disorders of pregnancy are limited particularly in Ethiopia. The aim of the study was to assess risk factors associated with hypertensive disorders of pregnancy among mothers in public hospitals of Tigray.

**Methods:**

The study was conducted in seven public hospitals of Tigray region, Ethiopia from June 2017 to November 2017. A facility based matched case-control study was employed to select 110 cases and 220 controls who were pregnant women. Cases and controls were matched by parity status. A case was a mother diagnosed to have hypertensive disorders of pregnancy by an obstetrician in the antenatal period while a control was a mother who did not have a diagnosis of hypertensive disorders of pregnancy. Data were collected by face to face interview technique using a pretested questionnaire and a checklist. Conditional logistic regression analysis was used to identify the independent predictor variables. Adjusted matched odds ratio with its corresponding 95% confidence interval was used and significance was claimed at *P*-value less than 0.05. Overall findings were presented in texts and tables.

**Results:**

Rural residents were at greater odds of suffering from hypertensive disorders (OR = 3.7, 95% CI; 1.9, 7.1). Similarly, mothers who consume less amount of fruits in their diet had 5 times higher odds of developing hypertensive disorders than those who consume fruits regularly (OR = 5.1, 95% CI; 2.4, 11.15). Overweight (BMI > 25 Kg/m2) mothers were also at risk of developing hypertensive disorders of pregnancy as compared with the normal and underweight mothers (AOR = 5.5 95% CI; 1.12, 27.6). The risk of developing hypertensive disorders of pregnancy was 5.4 times higher among diabetic mothers.

**Conclusion:**

Rural residence, less fruit consumption, multiple pregnancy, presence of gestational diabetes mellitus and pre-pregnancy overweight were identified as independent risk factors in this study. It is recommended that health care givers may use these factors as a screening tool for the prediction, early diagnoses as well as timely interventions of hypertensive disorders of pregnancy.

**Electronic supplementary material:**

The online version of this article (10.1186/s12884-018-2106-5) contains supplementary material, which is available to authorized users.

## Background

According to the American college of obstetricians and gynaecologists (ACOG), Hypertension in pregnancy is defined as: Systolic blood pressure greater than or equal to 140 mmHg and/or diastolic blood pressure greater than or equal to 90 mmHg in two occasions at least 6 h apart after fifth month of gestation for pregnancy induced hypertension or before pregnancy/before 20 weeks of gestation for chronic hypertension. Hypertensive disorders of pregnancy (HDP) refers to categories of conditions characterized by elevated blood pressure and classified as chronic hypertension (of any cause diagnosed before 20 weeks of gestation), gestational hypertension, chronic hypertension with superimposed preeclampsia and preeclampsia –eclampsia [1, 2].

Hypertensive disorder of pregnancy is one of the most common complications in pregnancy forming a triad together with hemorrhage and infection. It affects about 10% of pregnancies [3] and contributes for a significant maternal and perinatal mortality [4]. The World Health Organization (WHO) reported that 14.0% of global maternal deaths are attributed to hypertensive disorders of pregnancy [5]. In Latin-American and Caribbean countries 25.7% of maternal deaths were due to hypertensive disorders of pregnancy; in Asian and African countries, it contributed to 9.1% of maternal deaths and in fact about 16% in sub-Saharan African countries [5–7].

Hypertensive disorder of pregnancy is a global public health concern both in developed and developing countries. However, the risk that a woman in a developing country will die of the complications of hypertensive disorders of pregnancy is approximately 300 times higher than that for a woman in a developed country. A woman who develops pre-eclampsia is three times more likely to progress to eclampsia and if eclampsia is developed it is up to 14 times more likely to die of eclampsia [8].

The Ethiopian National Emergency Obstetric and Newborn Care (EMONC) study showed that pre-eclampsia/eclampsia complicated 1.2% of all institutional deliveries. Besides, 11% of all maternal deaths and 16% of direct maternal deaths were due to this obstetric complication [9] in another study in Ambo, Ethiopia maternal mortality due to hypertensive disorders of pregnancy was reported to be 12.3% [10] . The Ethiopian government has implemented different strategies to improve maternal health through increasing demand for services and easier access to emergency obstetric services. Expansion of health facilities, increased availability of supplies and deployment of appropriately skilled health professionals were among the strategies [11].

Despite the extensive research conducted the exact etiology of hypertensive disorders of pregnancy remained obscure. Thus, it is called a “disease of theories.” It is a multisystem disease with a heterogeneous nature and variable progression [12]. It has been proposed that immunological, nutritional and genetic factors as well as vascular and inflammatory changes are contributing for the development of hypertensive disorders of pregnancy [4].

Cognizant that the disease has no definite cause, several studies focusing on risk factors have been conducted in different parts of the globe and identified various risk factors for hypertensive disorders of pregnancy. These risk factors include socio-demographic variables such, personal and lifestyle factors, obstetric related factors, familial factors and medical related variables [13–16]. Specifically, nulliparity, extreme ages, obesity, a family history of hypertension, previous history of hypertensive disorders of pregnancy in multipara women, personal/family history of chronic hypertension/diabetes mellitus, high energy diet, gestational diabetes, mental stress during pregnancy, long inter-pregnancy interval, lower socioeconomic status and inadequate antenatal supervision were found to be associated with higher risk of developing hypertensive disorders of pregnancy in most studies [17–22]. Studies identified rural residence as a risk factor [23] and taking fruit or vegetables during pregnancy were found to be protective of hypertensive disorders of pregnancy [19].

Generally, maternal mortality due to hypertensive disorders of pregnancy remained high in spite of all the efforts. Studies conducted in different parts of the globe reported a range of risk factors though findings were not conclusive showing variations among populations and ethno-geographic groups. Moreover, inconsistent findings prevail across literatures even for a particular risk factor. Besides, there is paucity of evidence regarding factors associated with hypertensive disorders of pregnancy in Ethiopia. Even the few published studies conducted in Ethiopia were based on a document review which might have introduced bias due to incompleteness and poor quality of the data at the health facility [24, 25]. Thus, the current study attempted to assess risk factors for hypertensive disorders of pregnancy in Tigray region to generate evidences which are most relevant to support health policies and strategies.

## Methods

### Study setting and period

This study was conducted in selected public hospitals in Tigray region. Seven hospitals were included in the study namely Ayder, Mekelle, Adigrat, St. Marry, Suhul, Lemlem Carl and Kahsay Abera hospital. The six hospitals are located at the centre of the six respective zones of Tigray region mainly serving the people of the zones. Ayder referral hospital is found in Mekelle city serving as a referral hospital for about 8 million people from the entire Tigray region and partly from Afar and Amhara regions. In Tigray region, there are 28 health facilities providing basic emergency obstetrics and newborn care (BEmONC) and 15 facilities providing comprehensive emergency obstetrics and newborn care respectively [26]. The selected hospitals provide services for substantial number of patients with and without obstetrics complications. These hospitals are selected in this study due to the fact that they are staffed by obstetricians who can correctly diagnosed hypertensive disorders of pregnancy and relatively equipped by diagnostic facilities. Data were collected from June 2017 to November 2017.

### Study design

A facility based matched case control study was employed. These women were pregnant mothers attending antenatal care clinics in the study hospitals. Cases and control were matched in parity, time and site of the study. The case and control mothers were included after 20 weeks of gestation as per the diagnosis and the criteria set.

### Study population

The study population were all pregnant mothers attending the maternity centers of the study hospitals. Mothers with a history of confirmed chronic hypertension or diagnosed before 20 weeks gestation which is greater than or equal to 140/90 mmHg and without superimposed preeclampsia were excluded from the study because chronic hypertension can be a risk factor for preeclampsia but not for gestational hypertension. Since we measured the different hypertensive disorders as a single outcome, chronic hypertension was excluded as it can be an outcome and a risk factor at the same time. Chronic hypertensive women superimposed with preeclampsia-eclampsia was included as an outcome because this category has common exposure as the rest of the categories.

A case was defined as a mother diagnosed to have hypertensive disorders of pregnancy by an obstetrician in the antenatal period (*international classification of disease*/ICD − 10 codes O13, O14 and O15 [27]). Hypertensive disorders of pregnancy included gestational hypertension, preecalampsia-eclampsia and preeclampsia/eclampsia superimposed on chronic hypertension.

A control was defined as a pregnant women enrolled in the antenatal care clinic of the hospital and who did not have a diagnosis of hypertensive disorders. For each case two controls were interviewed in the same day and the same facility where the case was identified. Besides, cases and controls were matched according to their parity category.

### Sample size determination and sampling procedure

The sample size was calculated based on the comparison of proportions for matched case-control study using the following assumptions: Considering 95% CI, 80% power, case to control ratio of 1:2 and taking different sample size were produced for different risk factor for hypertensive disorders of pregnancy. Maximum sample size was obtained taking History of paternal hypertension as a risk factor from a previous study in Cameroon [28] where the proportion of exposure among cases to be 17.4% and among controls, 6%. Accordingly, these yields a maximum sample size of 100 cases and 200 controls. Adding a 10% non-response rate, the final sample size required for the study was 110 cases and 220 controls.

All cases who fulfil the defined criteria were consecutively included until the desired sample size was obtained. For every case included, two controls who best matched were identified.

### Operational definitions [2]

Hypertensive disorders of pregnancy- mother diagnosed with gestational hypertension, preeclampsia-eclampsia, chronic hypertension with superimposed preeclampsia or chronic hypertension (of any cause).

Gestational hypertension- systolic blood pressure ≥ 140 mmHg and/or diastolic blood pressure ≥ 90 mmHg measured on two occasions at least 4 h apart after twenty weeks of gestation in the absence of proteinuria or other systemic symptoms.

Preeclampsia- characterized by new onset of hypertension after 20 weeks gestation (systolic blood pressure ≥ 140 mmHg and/or diastolic BP ≥90) mmHg and proteinuria. However, in the absence of proteinuria other manifestations such thrombocytopenia (platelet count less than 100,000/μl), impaired liver function (elevated blood levels of liver transaminases to twice the normal concentration), the new development of renal insufficiency (elevated serum creatinine greater than 1.1 mg/dl or a doubling of serum creatinine in the absence of other renal disease), pulmonary edema, or new onset cerebral or visual disturbances are used to diagnose the case.

Eclampsia- characterized by new onset grand mal seizures in a woman with preeclampsia.

Chronic hypertension- includes essential hypertension as well as hypertension secondary to a range of conditions which is characterized by a blood pressure greater than or equal to 140 mmHg systolic and/or 90 mmHg diastolic confirmed before pregnancy or before 20 completed weeks gestation.

Chronic hypertension superimposed with Preeclampsia - mothers known to have hypertension before pregnancy or before 20 weeks of gestation and who had developed signs of preeclampsia after 20 weeks of gestation.

Proteinuria- a dipstick result of 1+ and above in a qualitative measurement.

### Data collection

Data collection was carried out in the maternity ward (antenatal care clinic and labor and delivery ward). It was collected by face to face interview technique using a pretested questionnaire. The questionnaire was developed following a thorough review of literatures from different sources and it included information related to socio-demographic condition, obstetrics and medical status, lifestyle and nutritional habits of the participants (Additional file 1). In addition to the questionnaire, patient medical records were reviewed to abstract relevant variables related with laboratory, clinical and obstetrics data. It was conducted by trained midwives and supervised by MPH professionals.

### Measurement

Height was measured in standing position bare foot and expressed in centimetres while weight was recorded in killograms. Body mass index (BMI) was calculated as weight (pre-pregnancy weight in the preceding 3 months) divided by height in meter square (kg/m^2^). For those who failed to remember their pre-pregnancy weight, the measured weight in the first trimester was taken as the weight gain during this time is low. In areas where women do not book early for antenatal care as the case in developing countries, pregnancy BMI is not recommended. Hence, in this case the pre-pregnancy weight was considered to calculate body mass index. In addition maternal mid-upper arm circumference (MUAC) was measured as it is considered to be relatively stable during pregnancy [29].

Maternal height category was made according to the calculated percentiles of the study participant and classified into four groups: ≥160 cm (25th percentile and lower); 161–162 cm (26th to 50th percentile); 163–165 cm (51th to 75th percentile); and ≥ 166 cm (76th percentile and higher). Body mass index (BMI) defined as pre-pregnancy weight in kilogrammes divided by height in meters squared, was categorised as follows: underweight (BMI < 18.5); normal weight (BMI = 18.5–24.9); overweight (BMI = 25–29.9); and obese (BMI ≥30). Likewise, monthly income was categorized into the lowest 25 percentile (below $91.9), between 25 and 75 percentile ($92–183.7), and above 75 percentile (greater than $183.8). Harvard university food frequency questionnaire [30] was used to assess the fruit and vegetables consumption status of mothers. Accordingly to the FFQ dietary assessment a list of fruits and vegetables were offered and asked how often they eat on average with in the last one year (ranging from never or less than once per month to 6+ per day). Those women who consumed fruits more than 2–4 times per week were considered as regular consumers of fruits or vegetables. For coffee consumption both frequency and volume were assessed.

### Data quality control

The questionnaire was prepared in English and translated into Tigrigna and back to English by independent language experts for consistency. Pre-test was conducted ahead of the actual data collection to see the appropriateness of the tool. Three days training was given for data collectors and supervisors on the content of the questionnaire and its administration. In order to maintain data quality primary data were collected from participants prospectively. The supervisors and the principal investigator checked questionnaires for completeness and inconsistencies on a daily basis.

### Analysis

Data entry was done in EPI-info 7 and exported to STATA Version 14 for cleaning and analysis; data cleaning was also done. Descriptive summary measures are reported. To identify factors associated with hypertensive disorders of pregnancy bivariate and matched analysis was done for the outcome of interest by comparing the cases with controls. Moreover, crude matched odds ratio and their 95% confidence intervals along with their *p* values in conditional logistic regression were calculated. In multivariable analysis, matched analysis was performed using conditional logistic regression to identify risk factors of hypertensive disorders. Adjusted odds ratio and their 95% confidence intervals were reported. Significance was declared at *P*-value ≤ 0.05. Multi-collinearity was checked among the independent variables by running the *regress* and *vif* syntaxes in the *stata* software. Accordingly, the variance inflation factor (*VIF*) was close to one and the tolerance which is the reciprocal of the variance inflation factor was also far above 0 which showed minimal collinearity. Post estimation command (Hosmer and Lemeshow test) in the logistic regression was run by using the *estat gof* to check the model fitness. Thus, the *p*-value for the Hosmer and Lemeshow chi-square was greater than 0.05 which indicated the fitness of the model. Overall findings were presented in texts and tables.

### Ethical consideration

The study was approved by the institutional review board of the college of health sciences Addis Ababa University. Participants involved in the study voluntarily. There were no other risks for the participants to participate in the study, other than those encountered in day-to-day life. It was described that information obtained from this study may be of valuable to mothers and new-borns in general. The anonymity of the study was maintained by excluding personal identifiers from the data collection tool and the records of the study were kept strictly confidential. Finally Informed consent was sought from the participants.

## Results

### Socio-demographic characteristics

A total of 330 mothers were interviewed in the data collection period that was held from June to November, 2018. Overall 110 cases matched on parity, day of interviews and study site/hospital with 220 controls taken part in the study to identify risk factors of hypertensive disorders of pregnancy. Of the total cases, gestational hypertension, preeclampsia, eclampsia and preeclampsia/eclampsia superimposed on chronic hypertension comprised of 36(32.7%), 55(50%), 14(12.7%), and 5(4.5%) respectively. Respondents were predominantly married, Orthodox Christianity followers and Tigrian by ethnicity in both cases and controls (90% and above in all cases). Regarding the occupation, majority of the mothers were housewives and comparable proportions were reported among cases and controls (64.5% Vs 68.2%). The Mean ± (SD) age of cases and controls were 27.6 ± 5.6 and 26.7 ± 5.8 years respectively. The proportion of older age mothers (age ≥ 35) was found to be higher among cases as compared to controls (23.6% Vs 11.8%)(*P* = .006). Besides, rural residents were higher among cases 71(64.5%) as compared to controls 76 (34.5%) (*P* < .001) (Table 1).

**Table 1:** Socio-demographic characteristics of mothers with and without hypertensive disorders of pregnancy in Tigray, 2018

variable	HDP/Cases *N* = 110, N (%)	No HDP/Controls *N* = 220, N (%)	COR (95% CI)	*P*-value
Age group				
≤ 18	7(6.4)	10(4.6)	1.5(0.58, 4.1)	0.378
19–34	77(70.0)	184(83.6)	1.0	
≥ 35	26(23.6)	26(11.8)	2.3(1.3, 4.2)	0.006
Residence				
rural	71(64.5)	76(34.5)	3.1 (1.9, 5.0)	< 0.001
urban	39(35.4)	144(65.4)	1.0	
Marital status				
married	104(94.5)	199(90.5)	1.0	
Unmarried	6(5.4)	21(9.5)	0.5 (0.2,1.4)	0.2
Partner change				
Yes	14(12.7)	26(11.8)	1.09 (0.5, 2.2)	0.8
No	96(87.2)	194(88.2)	1.0	
Religion				
orthodox	103(93.6)	199(90.5)	1.4 (0.5, 3.6)	0.49
Muslim	6(5.5)	16(7.3)	1.0	
Maternal education				
literate	74(67.3)	153(69.5)	1.0	
illiterate	36(32.7)	67(30.5)	1.12(0.7, 1.9)	0.65
Ethnicity				
Tigrian	105(95.5)	200(90.9)	1.0	
Amhara	4(3.6)	17(7.7)	0.4 (0.14, 1.36)	0.15
Occupation				
Housewife	71 (64.5)	150(68.2)	1.0	
Government employee	22(20.0)	33(15)	1.4(0.7, 2.7)	0.26
NGO employee	8(7.3)	7(3.2)	2.5(0.8, 7.7)	0.08 0.17
Private employee	7(6.4)	28(12.7)	0.5(0.2,1.3)	
Husband education				
Illiterate	14(12.7)	31 (14.1)	0.9 (0.4, 1.9)	0.8
Read and write	14(12.7)	26(11.82)	1.1(0.5, 2.3)	0.7
Primary	23(20.9)	42(19.1)	1.1(0.6, 2.1)	0.6
Secondary and above	59(53.6)	122(55)	1.0	
Income category				
≤ 2500	37 (33.64)	62 (28.18)	0.8(0.4,1.5)	0.5
2501–4999	48 (43.64)	106 (48.18)	0.9 (0.5, 1.7)	0.8
≥ 5000	25 (22.73)	52 (23.64)	1.0	

### Dietary, familial and lifestyle factors

Twenty two (20%) of pregnant mother had family history of hypertension among cases while only 14(6.4%) pregnant women had family history of hypertension among controls. The mean pre-pregnancy weight of cases and controls were 53.6 ± 8.4 and 51.3 ± 6.8 Kg, respectively. The maximum BMI recorded was 29.9 kg/m^2^; 65 (59.1%) and 147 (66.8%) of the respondents had BMI ranging from 18.5 to 25 kg/m^2^ in cases and controls respectively. The mid-upper arm circumference of mothers was categorized below the mean and above the mean (≥22. 1 and > 22.1) centimeters and more than 60% of the cases and controls were measured less than or equal to the mean. On average, the pre-pregnancy BMI was higher in women with hypertensive disorders than in those with normal pregnancies (20.36 ± 3.0 Vs 19.8 ± 2.6) (*P* = .05). Vegetable and fruit use were found to be less frequent in hypertensive disorders of pregnancy as compared with the normotensive women (42.7% Vs 60.4 and 54.5% Vs 87.7%). Likewise, frequency and volume of coffee use was demonstrated to be higher among cases when compared with controls (*P*=. 01, *P*=. 03) (Table 2).

**Table 2:** Dietary, familial and lifestyle characteristics of mothers with/without hypertensive disorders of pregnancy in Tigray, 2018

variable	HDP/Cases *N* = 110, N (%)	No HDP/Controls *N* = 220, N (%)	COR (95% CI)	*P*-value
Family history of hypertension				
Yes	22(20)	14(6.4)	3.6(1.7,7.6)	0.001
No	88(80)	206 (93.6)	1.0	
Mean weight ± (SD)	63.2 (8.7)	60.8 (7.3)		0.01
Mean Height ± (SD)	1.61(.06)	1.62(.05)		0.09
MAUC				
≤ 22.1	69 (62.7)	140(63.6)	1.0	
> 22.1	41(37.3)	80 (36.4)	1.0(0.6, 2.0)	0.82
Pre-pregnancy mean weight ± (SD)	53.6 (8.4)	51.3 (6.8)		0.006
Pre-pregnancy mean BMI ± (SD	20.36(3.0)	19.8 (2.6)		0.05
Fruit use				
Yes	60(54.5)	193(87.7)	1.0	
No	50 (45.5)	27 (12.3)	5.3 (3.0, 9.4)	< 0.001
Vegetable use				
Yes	47(42.7)	133(60.4)	1.0	0.002
No	63(57.3)	87(39.6)	2.1(1.3, 3.3)	
BMI of mothers				
< 18.5	33 (30.0)	67 (30.5)	1.0	
18.5–24.9	65 (59.1)	147 (66.8)	0.9(0.6,1.6)	0.8
≥ 25	12 (10.9)	6 (2.7)	4.3(1.4,13.6)	0.01
Coffee use				
Yes	93(84.5)	149(67.7)	3.0(1.6, 5.9)	0.001
No	17(15.5)	71(32.3)	1.0	
Frequency of coffee use (*N* = 242)				
≥ once a day	76 (81.7)	104 (69.8)	3.2 (1.3, 8.3)	0.01
< once a day	17 (18.3)	45 (30.2)	1.0	
volume of coffee use (*N* = 242)				
< 3 cups	28(30.1)	69(46.3)	1.0	
≥ 3 cups	65(69.9)	80(53.7)	2.1(1.0, 4.1)	0.03

### Obstetrics and medical factors

The proportion of multiple pregnancy was 16.4% among cases, while it was 4.5% among controls (*p* = 0.001). On the other hand, average age at menarche was reported to be 15 years, which were similar among cases and controls. About 3% of study participants had gestational diabetes mellitus and the proportion was different between cases and controls. It was 3.63% in cases while in controls it was 1.4% (*P* = 0.02) (Table 3).

**Table 3:** Obstetrics and medical characteristics of mothers with/without hypertensive disorders of pregnancy in Tigray, 2018

Variable	HDP/Cases *N* = 110, N (%)	No HDP/Controls *N* = 220, N (%)	COR (95% CI)	*P*-value
Pregnancy type				
Multiple	18 (16.4)	10 (4.5)	4.1(1.8, 9.6)	0.001
Single	92 (83.6)	210 (95.5)	1.0	
Gestational diabetes mellitus				
Yes	7 (6.36)	3 (1.4)	4.7(1.2,18.0)	0.02
No	103 (93.6)	217 (98.6)	1.0	
Pre-pregnancy oral contraceptive use				
Yes	42(38.2)	63 (28.6)	1.5 (0.9, 2.4)	0.08
No	68 (61.8)	157 (71.4)	1.0	
Presence of anemia at first visit				
Yes	94 (85.5)	194(88.2)	1.3 (0.6, 2.9)	
No	16(14.5)	26(11.8)	1.0	0.43
Age at menarche				
≤ 15 years	72 (65.4)	148 (67.3)	0.9(0.6, 1.5)	0.734
> 15 years	38 (34.6)	72 (32.7)	1.0	
Pre-pregnancy interval (*N* = 216)				
< 5 years	54 (83.08)	117 (77.5)	1.0	
≥ 5 years	11 (16.92)	34 (22.5)	0.9 (0.4, 2.2)	0.88
History of abortion				
Yes	25 (22.7)	39 (17.7)	1.0	0.263
No	85 (77.3)	181 (82.3)	0.7 (0.4,1.2)	

### Risk factors of hypertensive disorders of pregnancy

Bivariate analysis was run in the conductional logistic regression considering the discordant pairs between cases and controls to check the association between dependent and independent variables. Accordingly, rural residence, age > = 35 years, family history of hypertension, infrequent use of vegetables/fruits, higher pre-pregnancy weight, body mass index, coffee use, gestational diabetes mellitus and pre-pregnancy oral contraceptive use were identified as risk factors. In contrast, There was no difference among cases and controls with regard to average age, marital status, religion, ethnicity, occupation, maternal educational level, husband’s educational level, income, history of abortion, history of smoking, pre-pregnancy interval and age at menarche (Table 4).

**Table 4:** Bivariate and multivariable analysis for the predictors of hypertensive disorders of pregnancy in Tigray, 2018

Variables	Matched unadjusted OR(95% CI)	Matched adjusted OR(95% CI)
Residence		
Rural	3.1 (1.9, 5.0)*	3.7(1.9, 7.1)**
Urban	1.0	1.0
Age		
Mean ± (SD)	1.02(0.9, 1.06)	0.96 (0.9, 1.02)
Marital status		
Married	1.0	1.0
Unmarried	0.5(0.2, 1.4)	0.44 (0.12, 1.5)
Family History of hypertension		
Yes	3.6 (1.7, 7.6)*	2.1 (0.7, 6.4)
No	1.0	1.0
Fruit use		
Yes	1.0	1.0
No	5.3 (3.0, 9.4)*	5.1 (2.4, 11.15)**
Vegetable use		1.0
Yes	1.0	1.2(0.6, 2.3)
No	2.08 (1.3, 3.3)*	
History of smoking		
Yes	1.0	1.0
No	0.3 (0.07, 1.2)	0.6 (0.07, 5.2)
BMI of mothers (prepregnancy)		
< 18.5	1.0	1.0
18.5–24.9	0.95 (0.56, 1.6)	1.7 (0.8, 3.4)
25–29.9	4.3 (1.4, 13.6)*	5.5 (1.12, 27.6)*
Coffee use		
Yes	3.08 (1.6, 5.9)*	1.9 (0.8, 4.4)
No	1.0	1.0
Pregnancy type		
multiple	4.1 (1.8, 9.6)*	4.2 (1.3, 13.3)*
single	1.0	1.0
Presence of gestational diabetes mellitus		
Yes	4.6 (1.2, 18.0)*	5.4 (1.1, 27.0)*
No	1.0	1.0
Oral contraceptive use		
Yes	1.5 (0.9, 2.4)	1.2(0.6, 2.4)
No	1.0	1.0

*P-value <0.01, **P-value <0.001

Variables which were found to be associated with the outcome variable in the bivariate analysis (*P* < =0.2) were taken to the multivariable analysis. This is basically to compensate for the power of the test since negative findings (that is, *p* > 0.05) may be just because of inadequate power. After adjusting for possible confounding factors in the matched pair conditional logistic regression only residence, fruit use, pre-pregnancy BMI of mothers, types of pregnancy and gestational diabetes mellitus were found to be independent predictors of hypertensive disorders of pregnancy. Mothers who live in a rural area were at greater odds of having hypertensive disorders as compared to mothers who reside in urban area (OR = 3.7, 95% CI; 1.9, 7.1). Similarly, mothers who do not consume at all or consume less amount of fruits in their diet had 5 times higher odds of developing hypertensive disorders than those who consume fruits regularly (AOR = 5.1 95% CI; 2.4, 11.15). Overweight (BMI > 25 Kg/m^2^) mothers were also at risk of developing hypertensive disorders of pregnancy as compared with the normal and underweight mothers (AOR = 5.5 95% CI; 1.12, 27.6). In addition, multiple pregnancy and presence of diabetes mellitus were independent risk factors for the development of hypertensive disorders of pregnancy; the risk of developing hypertensive disorders of pregnancy was 5.4 times higher among diabetic mothers compared with those who are free of the disease (AOR = 5.4, 95% CI; 1.1, 27.0). On the other hand, the effect of age, family history of hypertension, use of vegetables, and drinking coffee disappeared in the multivariable analysis when adjusted for possible confounders.

## Discussion

The current study result showed that rural residence was associated with the development of hypertensive disorder of pregnancy. This finding is consistent with a previous finding in an epidemiological study among pregnant mothers in Cairo, Egypt [23]. This could be due to the fact that mothers from rural areas book antenatal care later in pregnancy and have fewer ANC visits which could be associated with delay in health seeking behaviour. This delay in health care seeking could in turn be influenced by lack of awareness on pregnancy related problems, husband and family influences, local cultural influence and bad experiences in health facilities.

Similarly fruit consumption was found to be important predictor in this study, mothers who consume less fruits in their diets were at higher risk of developing hypertensive disorders of pregnancy which is in line with previous findings reported from Bahrdar, Ethiopia ([19], Cairo, Egypt [23] and Norway [17]. This was also supported by a systematic review and meta-analysis of studies whereby calcium intake was found to be protective to hypertensive disorders of pregnancy in a multivariable analysis [31]. Fruits are rich in micronutrients and many of the vitamins and minerals play antioxidant role which could in turn help in the prevention of hypertensive disorders of pregnancy.

Pre-pregnancy body mass index was calculated and overweight mothers were at higher odds of developing hypertensive disorders of pregnancy as compared with low and normal body mass index which is in agreement with reports from USA [32, 33]. Likewise, multiple pregnancy has been reported as an independent predictor of hypertensive disorders of pregnancy from various studies in different parts of the globe [22, 34, 35]. The current finding is also in support of those previous reports which showed 4.2 times increased risk of developing hypertensive disorders of pregnancy compared with the singleton pregnancy.

Gestational diabetes mellitus was also found to be an independent predictor of hypertensive disorders of pregnancy that supported the existing knowledge; because literatures noted that pregnant mother who developed diabetes mellitus would have higher predisposition to develop hypertensive disorders of pregnancy and it has been identified as the most common predictor in previous studies [22, 34, 36–38].

Family history of hypertension was a predictor in the bivariate analysis but its effect vanished in the adjusted model and this contradicts with previous reports. These studies reported an increased risk of hypertensive disorders with a positive family history of chronic hypertension [21, 28, 36, 39–41].

Similarly, drinking more than 3 cups of coffee per day was not a significant risk factor in this study which means it is in conformity with some studies showing no difference [42] and contradicted with others. For instance, a study in Bahrdar, Ethiopia showed that mothers who reported to have taken coffee during pregnancy had higher odds of developing preeclampsia [19]. However, another study in Rotterdam, the Netherlands reported the substantial protection of coffee against the development of pregnancy induced hypertension [42].

Extreme lower or higher ages in pregnancy (age < 20 and > 35 years) were reported as a risk factor for hypertensive disorders of pregnancy in previous studies; *Tebeu PM et.al* reported that teenage mothers were at increased risk of developing hypertensive disorders [28] on the other hand, *Suzuki. S.* and *Igarashi M.* in their study revealed that age > = 35 was a significant factor for the development of preeclampsia [43] but in the current study though age > = 35 showed a significant risk in the first model, no difference was observed in the adjusted model. The difference may be due to the fact that majority of the respondents were within the age range of 19–34.

In many studies nulliparity was reported as a common risk factor for the development of hypertensive disorders of pregnancy [32–34, 41] but in this study its effect was not possible to measure as it was a matching variable. Unlike the current finding, partner change was reported as a risk factor for hypertensive disorders of pregnancy in other literatures [23]. The reason may be there were few mothers who changed their partner in the study and this in turn could make the difference invisible.

In previous studies illiteracy was reported to be a risk factor for hypertensive disorders of pregnancy [28] as it affects the age at marriage and pregnancy as well as health seeking behaviour but in the current study no association was reported. The continuous health education program provided by the health extension workers at the community and household levels might have helped to have similar level of awareness about the issue.

Some studies reported inter-pregnancy interval as a risk factor for hypertensive disorders of pregnancy. Longer inter-pregnancy interval had higher risk of developing hypertensive disorders of pregnancy [44] but in the current study no association was found.

The aforementioned findings should be viewed in light of the following limitations. Since cases were selected consecutively as soon as they were identified, selection bias might be introduced. Moreover, dietary assessment was self-reported and assessed at diagnosis which could have introduced recall bias.

## Conclusion

The study assessed different risk factors of hypertensive disorders of pregnancy. Thus, rural residence, less fruit consumption, multiple pregnancy, presence of gestational diabetes mellitus and pre-pregnancy overweight were identified as independent risk factors. This highlights that there is a need to extend obstetric services to the grass root level in which rural residents can get all types of services in a closer distance. In addition, it necessitates strong nutritional education for the community during pregnancy and even the time preceding pregnancy including the routine supply of supplements. There is also a need to remind health professionals to properly identify and manage pregnant women having diabetes mellitus. It is recommended that these factors can be used as a screening tool for the prediction, early diagnoses as well as timely interventions of hypertensive disorders of pregnancy.

## Additional file

Additional file 1: The tool used to collect the information (caes-control study) has been attached as additional file. (DOCX 30 kb)

## Abbreviations

ACOG: American College of Obstetricians and Gynaecologists
BEmONC: Basic Emergency Obstetrics and Newborn Care
BMI: Body Mass Index
EmONC: Emergency Obstetric and Newborn Care
HDP: Hypertensive Disorders of Pregnancy
ICD: International Classification of Disease
MPH: Master of Public Health
MUAC: Mid Upper Arm Circumference
WHO: World Health Organization
