# Supplementary material for: Risk factors for hypertensive disorders of pregnancy among mothers in Tigray region, Ethiopia: matched case-control study
Source: BMC Pregnancy Childbirth. 2018 Dec 6;18:482. doi: 10.1186/s12884-018-2106-5 (PMC6282279; doi:10.1186/s12884-018-2106-5)
Supplement: Supplementary file 1 — The tool used to collect the information (caes-control study) has been attached as additional file. (DOCX 30 kb) [file 12884_2018_2106_MOESM1_ESM.docx]

**Information sheet and consent form**

**Title:** Assessment of risk factors of hypertensive disorders of pregnancy among mothers in selected hospitals of Tigray

**Introduction**

Good Morning/Good Afternoon

My name is ………………………………………, from Addis Ababa university college of health sciences school of public health carrying out a research on ‘the assessment of risk factors of hypertensive disorders of pregnancy among mothers in selected hospitals of Tigray. I am interviewing pregnant mothers here whose gestational age is 20weeks and above; you are also included in this study because you are pregnant mother. To attain its purpose, your honest and genuine participation by responding to the question prepared is very important & highly appreciated. We ask that you read this form and ask any questions that you may have before agreeing to participate in the study.

**PURPOSE OF STUDY**

The purpose of the study is to identify the different types of risk factors for developing various kinds of hypertensive disorders in mothers. At the end we will be able to unveil the significant risk factors associated with the development of hypertensive disorders and recommendations will be given so as to improve the management and reduce morbidity and mortality associated with hypertensive disorders of pregnancy. Ultimately, this research will be published in national and international journals for wider visibility and usage.

**STUDY PROCEDURES**

If you agree to be in this study, you will be asked regarding your socio-demographic characteristics and about your usual activities in your everyday life. The interview will take about 20 to 25 minute. Again I am very much appreciating your participation in this interview.

**VOLUNTARY PARTICIPATION**

Your participation in this study is voluntary. It is up to you to decide whether or not to take part in this study. If you decide to take part in this study, you will be asked to sign a consent form. After you sign the consent form, you are still free to withdraw at any time and without giving a reason. Withdrawing from this study will not affect the relationship you have, if any, with the researcher or your decision will not result in any loss or benefits to which you are otherwise entitled. Additionally, you have the right to request that the interviewer not use any of your interview material.

**RISKS**

There are no reasonable foreseeable (or expected) risks to you for participating in this study other than those encountered in day-to-day life. Some questions may make you feel uneasy. You may not be familiar with some of the questions or issues. You can ask for elaborations on questions you think you do not properly understand.

**BENEFITS**

There will be no direct benefit to you for your participation in this study. However, we hope that the information obtained from this study may be of valuable to mothers and newborns in general.
**CONFIDENTIALITY**

This study is anonymous. We will not be collecting or retaining any information about your identity. The records of this study will be kept strictly confidential. Research records will be kept in a locked file, and all electronic information will be coded and secured using a password protected file; only the researcher will have access to the records. In any sort of report we make public we will not include any information that will make it possible to identify you.

**COMPENSATION**

No compensation for participating in this study. You will not receive any monetary or any other kind of compensation for participating in this interview.

**CONTACT INFORMATION**

You have the right to ask questions about this research study and to have those questions answered by me before, during or after the research. If you have any further questions about the study, at any time feel free to contact me, [data collector] by telephone at [*phone number*]. If you like, a summary of the results of the study will be sent to you. If you have any other concerns about your rights as a research participant that has not been answered by the data collector, you may contact **Hailemariam Berhe** the principal investigator by telephone at [*+251914707632*].

If you have any further problems or concerns that occur as a result of your participation, you can report them to the IRB at Addis Ababa University, college of health sciences.

**CONSENT**

I have read and I understand the provided information and have had the opportunity to ask questions. I understand that my participation is voluntary and that I am free to withdraw at any time, without giving a reason and without cost. I do also understand that there is no risk in participating in the study, so I voluntarily agree to take part in this study.

Data collector’s Name and signature______________________________Date _____________

**Participant’s status**

1. With hypertensive disorder **(case) 🞏**
2. Without hypertensive disorder **(control) 🞏**

Put **(**√**)** sign in the box above

| **No** | **Question** | **Response** | **Remark** |
| --- | --- | --- | --- |
| **1** | **Socio-demographic characteristics** | |  |
| 201 | How old are you? | ______(years) |  |
| 202 | Where do you live? | 1. Urban 2. Rural |  |
| 203 | What is your marital status | 1. Married 2. Single 3. Divorced 4. widowed 5. Separate |  |
| 204 | Have you got pregnant from your first partner for this index pregnancy? | 1. Yes 2. No | 1🡪206 |
| 205 | If the answer for Q. Number 204 is No, what is the order of the partner for this index pregnancy? | 1. 2^nd^ 2. 3^rd^ 3. 4^th^   99. others (specify)________ |  |
| 206 | What is your religion? | 1. Orthodox 2. Muslim 3. Catholic 4. Protestant 5. Others(specify)____________ |  |
| 207 | What is your Ethnicity? | 1. Tigraway 2. Amhara 3. Others (specify)____________ |  |
| 208 | What is your level of education? | 1. No education 2. read and write 3. primary 4. Secondary and higher |  |
| 209 | What is your occupation? | 1. Housewife 2. Government employee 3. Nongovernmental employee 4. Private Organization 5. Daily labourer 6. Other (specify)____________ |  |
| 210 | What is your spouse’s education level | 1. No education 2. read and write 3. primary 4. Secondary and higher |  |
| 211 | What is your monthly household in come | ______________Birr |  |
| **2** | **Familial and Life style variables** | | |
| 212 | Family history of hypertension (parents, father, grandparents and siblings) | 1. Yes 2. No |  |
| 213 | Weight | _______kg |  |
| 214 | Height | ____________m |  |
| 215 | MUAC | ___________cm |  |
| 216 | Have you ever smoked? | 1. Yes 2. No |  |
| 217 | Have you ever used traditional treatment for any problem developed during the index pregnancy? | 1. Yes 2. No | 2🡪221 |
| 218 | If the response to Q. No 219 is yes, specify the type of remedy taken? | __________________________________________________________________________ |  |
| 219 | What was your pre-pregnancy weight | ____________kg |  |
| 220 | Do you drink coffee (before pregnancy and now) | 1. Yes 2. No | 2🡪223 |
| 221 | If the response to Q No 222 is yes? How often do you drink at home and outside? | 1. More than once daily 2. Daily 3. 2-3 times a week 4. Once a week 5. Less than once a week   99. Others specify ____________ |  |
| 222 | If the response to Q No 221 is yes? How much do you drink at each episode at home and outside? | 1. 1 cup of coffee 2. 2 cup of coffee 3. 3 cup of coffee 4. More than 3 cup of coffee   99. Others specify ________ |  |
| **Obstetrics and medical related variables** | | | |
| 223 | Multiple gestation | 1. Yes 2. No 3. Unknown |  |
| 224 | Gestational diabetes mellitus | 1. Yes 2. No |  |
| 225 | Have you been using Combined oral contraceptive before you get pregnant? | 1. Yes 2. No |  |
| 226 | What was your age at menarche | _______years |  |
| 227 | Have you had hypertensive disorders of pregnancy in previous pregnancy | 1. Yes 2. No 3. I got pregnant for the first time | 3🡪229 |
| 228 | If the answer for Q. No 229 is 1or 2 what is the pregnancy interval between this pregnancy and the immediate previous delivery | _________ (years) |  |
| 229 | History of abortion | 1. Yes 2. No | 2🡪231 |
| 230 | If the answer for Q. No 231 is yes, how many times? | ___________ |  |
| 231 | Pre-gestational diabetes mellitus | 1. Yes 2. No |  |
| 232 | Do you have known co-morbidities  **Multiple response is possible** | 1. Anemia 2. Autoimmune disease 3. Cardia disease 4. Renal disease 5. Others_____________ |  |
| 233 | Presence of anemia at first visit | 1. Yes 2. No |  |
| 234 | Gestational age at diagnosis | ___________(weeks) | **For cases** |
| 235 | Type of hypertensive disorders of pregnancy | ________________________________________________________________________ |  |

For each food listed, fill in the circle indicating how often on average you have used the amount specified during the last year

| Fruits | Never, or less than once a month | 1-3 per month | 1 per week | 2-4 per week | 5-6 per week | 1 per day | 2-3 per day | 4-5 per day | 6+ per day |
| --- | --- | --- | --- | --- | --- | --- | --- | --- | --- |
| Banana |  |  |  |  |  |  |  |  |  |
| Avocado |  |  |  |  |  |  |  |  |  |
| Apples |  |  |  |  |  |  |  |  |  |
| Apples juice |  |  |  |  |  |  |  |  |  |
| Orange |  |  |  |  |  |  |  |  |  |
| Orange juice |  |  |  |  |  |  |  |  |  |
| Grape fruits |  |  |  |  |  |  |  |  |  |
| Papaya |  |  |  |  |  |  |  |  |  |
| Mango |  |  |  |  |  |  |  |  |  |
| Strawberries |  |  |  |  |  |  |  |  |  |
| Vegetable | | | | | | | | | |
| Tomatoes (juice, slice, sauce) |  |  |  |  |  |  |  |  |  |
| Cabbage |  |  |  |  |  |  |  |  |  |
| Carrots |  |  |  |  |  |  |  |  |  |
| Mixed vegetables |  |  |  |  |  |  |  |  |  |
| Sweet potatoes |  |  |  |  |  |  |  |  |  |
| Green mustard |  |  |  |  |  |  |  |  |  |
| Spinach |  |  |  |  |  |  |  |  |  |
| Lettuce |  |  |  |  |  |  |  |  |  |
| Pepper |  |  |  |  |  |  |  |  |  |
| Onions (including in salad) |  |  |  |  |  |  |  |  |  |

Source: HARVARD UNIVERSITY Dietary assessment tool
